# Diffuse Alveolar Hemorrhage in a Patient with Antisynthetase Syndrome

**DOI:** 10.1155/2019/5453717

**Published:** 2019-07-31

**Authors:** D. A. Vargas-Gutiérrez, F. Solís-Jiménez, K. I. Arias Callejas, L. Cano Cruz, R. Zapata Arenas, A. O. Acero López, P. Puente Rodríguez, S. Velázquez de la Paz

**Affiliations:** ^1^Internal Medicine Department, Hospital General de Mexico ‘Dr. Eduardo Liceaga', Mexico City, Mexico; ^2^Rheumatology Department, Instituto Nacional de Ciencias Médicas y Nutrición Salvador Zubirán, Mexico City, Mexico; ^3^School of Health Sciences, Anahuac University, Mexico City, Mexico; ^4^Mexican Medical School, La Salle University, Mexico City, Mexico

## Abstract

An alveolar hemorrhage case is reported as the initial manifestation of antisynthetase syndrome in a 40-year-old man, who is admitted to the Emergency Department for diagnostic approach of chronic cough and progressive dyspnea. The diagnosis of the alveolar hemorrhage was based on the presence of acute respiratory failure, decrease in hemoglobin levels, and observation of macrophages filled with hemosiderin. The antisynthetase syndrome was classified through a tomographic image compatible with a nonspecific interstitial pneumonia, along with antibodies associated to myositis (PL-12 and Ro-52). The study protocol was completed with the result of a myopathic pattern showed in electromyography. This patient presented a good response to steroids and disease-modifying antirheumatic drug (DMARD).

## 1. Introduction

Diffuse alveolar hemorrhage (DAH) is a clinical syndrome characterized by hemoptysis, the decrease of at least 2 grams of hemoglobin levels, hypoxemic respiratory failure, and the presence of new diffuse pulmonary infiltrates on a tomographic study. On the contrary, pulmonary capillaritis is defined as the infiltration of polymorphonuclear cells in septal and peribranchial vessels, generating capillaritis and disruption of the capillary membrane. Further accumulation of erythrocytes in alveolar spaces is observed. The histopathology includes intra-alveolar red cells, fibrin, and the accumulation of macrophages filled with hemosiderin [1, 2].

On a histopathology level, the most common underlying type of DAH is the pulmonary capillaritis, characterized by a small vessel vasculitis. Some main causes of capillaritis, listed according to their relation, are as follows: anti-neutrophil cytoplasmic antibody- (ANCA-) associated vasculitis, autoimmune connective tissue diseases, infectious disorders, and neoplasms. Current treatments focus on the underlying causes of the syndrome and include corticosteroids, immunosuppressive drugs, and seldom plasmapheresis [3].

As mentioned, the pulmonary diseases associated with myositis describe antibodies' mechanisms directed against numerous amino-tRNA synthetases. This is termed the “antisynthetase syndrome” (ASS). This syndrome occurs in conjunction with arthritis, myositis, and interstitial pneumopathy, most commonly known as “classic antisynthetase syndrome.” Some patients may present one or two of these manifestations, labelled as an “incomplete antisynthetase syndrome” [4]. In addition, other clinical findings are fever, Raynaud's phenomenon, and mechanic's hands (cracking and hyperkeratosis of the lateral surfaces of the fingers) [5].

The lung is the extra muscular organ most frequently involved in polymyositis (PM) and dermatomyositis (DM) descriptions. Organ complications occur in more than 40% of patients during their lifetime, contributing to annual statistics on morbidity and mortality [6]. In this context, respiratory failure or dyspnea is relatively uncommon, reported in less than 5% of patients with pulmonary involvement [5, 7]. However, damage to the pulmonary parenchyma caused by fibrosis can cause pulmonary arterial hypertension as well as acute alveolitis, both strongly linked to diffuse alveolar hemorrhage [8].

The aim of this report is to acknowledge the importance of alveolar hemorrhage as a manifestation of other pulmonary diseases and, in specific, of antisynthetase syndrome.

## 2. Clinical Case

**Figure 1:**
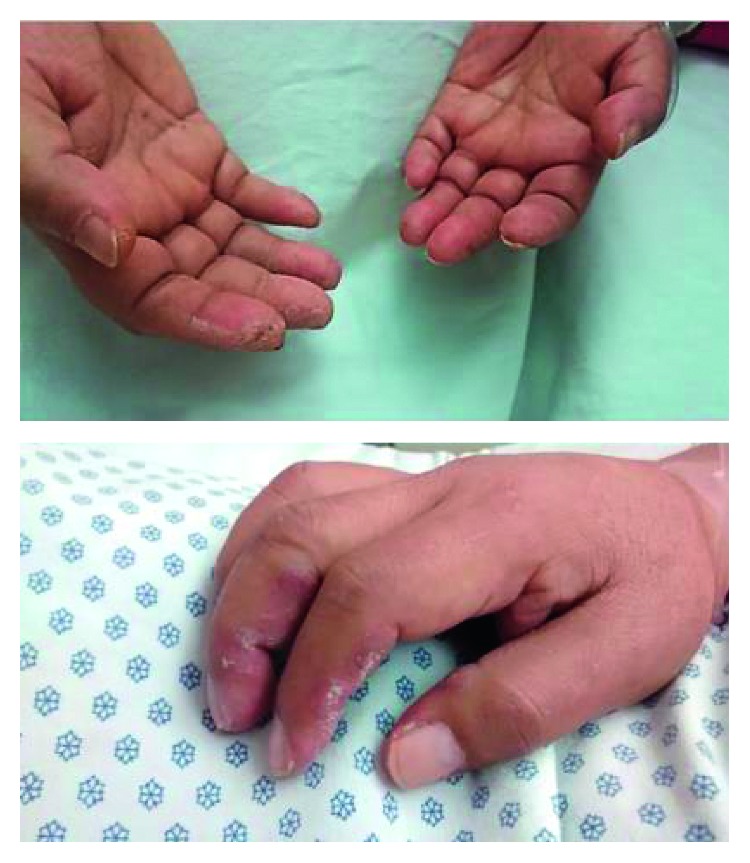
Hyperkeratosis on the lateral surfaces of the fingers.

A 40-year-old male patient, a lawyer from Mexico City, with no relevant chronic-degenerative background, presented with dry cough and moderate dyspnea for the last 8 months, which had gradually worsened. By this time, he had already been examined a couple of times and prescribed inhaled muscarinic bronchodilators, with partial improvement on the symptoms. Nonetheless, skin peeling on distal phalanges on both hands has emerged 15 days before his visit to the hospital, with no apparent trigger (Figure 1).

Evolving over time with functional class deterioration, the patient presents paroxysmal coughing and hemoptysis; hence, his hospital admission with clinical evidence of hypoxemia and moderate anaemia (hemoglobin 8.2 gr/dl).

**Figure 2:**
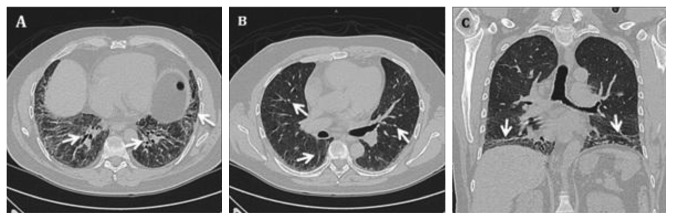
High-resolution computed tomography. (a) Bilateral pulmonary posterior basal sections showing a reticular pattern characterized by septal fibrosis and traction bronchiectasis. (b) At the carinal bifurcation region, a micronodular image is visible along with multiple subpleural ground-glass opacification zones. (c) Coronal plane evidence of fibrosis located in both lung bases.

Once hospitalized, the physical exam revealed jugular vein distention (JVD), preserved muscle strength, and subcrepitant rales in the subscapular region. Consequently, a pneumopathy research was initiated through a transthoracic echocardiogram (TTE), which showed a left ventricular concentric hypertrophy, preserved ejection fraction, and an increased pulmonary artery systolic pressure suggesting a moderate pulmonary hypertension. However, the patient persisted with respiratory deterioration, requiring invasive mechanic ventilation and further admission to the Intensive Care Unit (ICU), where he was initiated with an empirical antibiotic treatment. After 48 hours of showing a poor response to the antibiotic treatment, the staff decided to perform a high-resolution computed tomography, reporting a matching pattern of a nonspecific interstitial pneumonia (NSIP) (Figure 2).

Based on these findings, a bronchoalveolar lavage was performed showing the presence of loaded hemosiderin macrophages, confirming a diffuse alveolar hemorrhage; hence, a 1000 mg pulse IV methylprednisolone and cyclophosphamide therapy was established for 3 days.

A screening research for pauci-immune vasculitis was performed; nevertheless, organ damage and anti-neutrophil cytoplasmic antibodies (ANCAs) were not identified. Additionally, other causes of immune complex-mediated vasculitis were studied, but hepatitis B/C virus, tuberculosis, and HIV values were all negative and complement levels were normal. The sole finding that suggested that the patient was suffering from an autoimmune disease and was a strongly positive antinuclear antibodies ratio (1 : 1280), with cytoplasmic and ribosomal pattern, which implied to discard systemic lupus erythematosus. However, there was not enough evidence to justify a renal function deterioration, proteinuria, haematuria, or pyuria.

Although the patient did not meet the classification criteria for systemic lupus erythematous, the lookup for specific antibodies for myositis was conducted, highlighting an anti-PL-12 and anti-Ro-52 strongly positive result. Accordingly, subclinical myositis was confirmed through electromyography; results showed a recruitment pattern, even when the patient had never presented muscle enzyme elevation or decreased muscle strength in a significant manner. Conclusive diagnosis included incomplete antisynthetase syndrome and alveolar hemorrhage.

Once out of the intensive care unit, a prednisone 50 mg/day and methotrexate 15 mg/wk was followed for 3 months until the dyspnea and hemoptysis disappeared. Confirmed negative tests on neoplastic disease assured the patient's amelioration. One year later, the patient showed a considerable improvement of his respiratory function parameters. As a regular follow-up treatment, the patient is on low doses of methotrexate, practically asymptomatic.

## 3. Discussion

The DHA observed in this case is associated to a connective tissue disease, with specific electromyography of myositis, even though the presence of these two phenomena has been reported in literature as exceptional [9].

The patient did not show cutaneous wounds proper from dermal myositis, even though since he was admitted to the hospital, mechanical hands were identified. The classification criteria (EULAR/ACR 2017) do not consider mechanical hand as a criterion for dermal myositis (DM) [10]. Debates on this matter derived to an international meeting between 50 dermatologists and rheumatologists, held at the beginning of 2019. Through the Delphi method, they proposed a new classification where patients with DM were researched. The argument was well received among the team, therefore being acknowledged from then on as something to be considered on patients with regular evolution of classic DM [11, 12].

However, sinusoidal dermal myositis should be considered as a different diagnosis as it is a syndrome where weakness and histology defects are present, against as DM [13].

Chronic and repeated inflammation episodes generate pneumonitis which leads to accumulation of collagen in small ways of the respiratory tract and a potential fibrosis/interstitial pneumopathy [14]. The most common tomographic pattern associated with this antisynthetase syndrome is the nonspecific interstitial pneumopathy [15]. This pattern is the most compatible with the case here presented due to the glassy subpleural areas, with reticular patterns and traction bronchiectasis [16]. Even though these diffuse glassy areas allow alveolar hemorrhage to be easily identified, it can also be found in 20–50% of acute phases with no other clinical change related [17].

According to this, the patient did present the criterium from antisynthetase syndrome, evidenced by interstitial pneumopathies associated with serology for aminoacyl RNA synthetase (anti-PL-12) and the mechanical hands. Even not always related among each other, all confirmed to be linked with the described syndrome.

The antibodies specifically related to myositis are used as clinical biomarkers to diagnose PD/DM. Anti-Jo-1 is the main antisynthetase antibody identified in 15–25% of the patients. Additionally, diverse antibodies have been proposed for other 7 aminoacyl ARNt synthetases, with a prevalence of 1–5% and even less [18].

A retrospective study on Mexican population, including the one where the present patient grew up, was conducted so as to identify the main serological markers associated to inflammatory myositis. Results showed Mi-2 antibodies as the most frequent ones (35%), followed by Ro-52 (21%) and Jo-1 (4%) [19].

Anti-PL-22 antibodies guided against alanyl synthetase have been reported to 13.6%, with a clinical profile on black race and a major percentage of pulmonary involvement with a minimum muscular participation (also present in the patient) and without cancer and mortality correlations [20].

Anti-Ro-52 antibodies have been commonly related to NI and immune diseases, in particular, myositis and scleroderma. The prevalence of these patterns is 71.4%, against a negative result of 16.7%, with a statistical *p* of 0.018 [21].

Isabelle Marie and colleagues have studied 89 cases of patients with antisynthetase syndrome through the identification of Jo-1, where 32% reported positive Ro-52 values and 2.86 OR (IC 1.14–6.94) for mechanical hands and 4.46 OR (1.02–19.4) for malignant neoplasms. Accordingly, 75% of the patients were deceased after 72 months of treatment [21].

Idiopathic inflammatory myopathies have been associated with an increased risk of malignant neoplasms. This association can be better identified in dermal myositis where 2.15–6.5 times the risk is shown through studies. One of the most important and breakthrough improvements in this area has been the specific myositis antibodies which denote various clinical phenotypes: antibody anti-TIF 1 recognises a nuclear transcription protein and is present in 38–80% of patients developing malignancy at any point of their disease. Therefore, all patients with a recent diagnosis on myositis should be screened on malign neoplasia. Consequently, patients older than 18 with dermal myositis and TF-1 antibodies should be included for a screening on extension TAC/PET, gynaecological exploration, pelvic echography, mammography, and annual colonoscopy for 3 to 5 years. Presence of interstitial pneumopathy is considered a good forecast, reason why it has been proposed with and without malignant neoplasms [22].

## 4. Conclusion

In conclusion, although common etiologies of diffuse alveolar hemorrhage have been extensively classified, rare types of infrequent associations have neither place for identification nor exploration. Acknowledgement of their existence and their linking patterns must be reviewed and shared among the clinical research community in order to be able to enrich diagnosis and present classification criteria. The transcendence on identifying the correct origin of interstitial lung disease and diffuse alveolar hemorrhage lies on the establishment of wide therapeutic strategies that provide tools on diagnosis and treatment that benefit the patient.
